# Effects of a developmental dyslexia remediation protocol based on the training of audio-phonological cognitive processes in dyslexic children with high intellectual potential: study protocol for a multiple-baseline single-case experimental design

**DOI:** 10.1186/s12887-023-04189-6

**Published:** 2023-08-17

**Authors:** Gaëlle Darrot, Auriane Gros, Valeria Manera, Bruno De Cara, Sylvane Faure, Xavier Corveleyn, Karine Harrar-Eskinazi

**Affiliations:** 1 Département d’Orthophonie de Nice, Faculté de Médecine, Université Côte d’Azur, Nice, France; 2 Université Côte d’Azur, Laboratoire CoBTeK, Nice, France; 3 Université Côte d’Azur, Centre Hospitalier Universitaire de Nice, Laboratoire CoBTeK, Service Clinique Gériatrique du Cerveau Et du Mouvement, Nice, France; 4 Université Côte d’Azur, Laboratoire LAPCOS, Nice, France; 5 Hopitaux Pédiatriques de Nice CHU-LENVAL, Centre Hospitalier Universitaire de Nice, Nice, France

**Keywords:** Dyslexia, Gifted, Speech therapy, Underlying cognitive deficits, Study protocol

## Abstract

**Background:**

The significant prevalence of children with high intellectual potential (HIP) in the school-age population and the high rate of comorbidity with learning disabilities such as dyslexia has increased the demand for speech and language therapy and made it more complex. However, the management of dyslexic patients with high intellectual potential (HIP-DD) is poorly referenced in the literature. A large majority of studies on HIP-DD children focus on the screening and diagnosis of developmental dyslexia, but only a few address remediation. Developmental dyslexia is a severe and persistent disorder that affects the acquisition of reading and implies the impairment of several underlying cognitive processes. These include deficits in Categorical Perception, Rapid Automatized Naming, and phonological awareness, particularly phonemic awareness. Some authors claim that HIP-DD children's underlying deficits mainly concern rapid automatized naming and phonological awareness. Thus, the purpose of this study is to present a remediation protocol for developmental dyslexia in HIP-DD children. This protocol proposes to compare the effects on reading skills of an intensive intervention targeting categorical perception, rapid automatized naming, and phonemic analysis versus standard speech therapy remediation in HIP-DD children.

**Methods:**

A multiple-baseline single-case experimental design (A_1_BCA_2_) will be proposed to 4 French HIP-DD patients for a period of 30 weeks. Intervention phases B and C correspond to categorical perception training and rapid automatized naming training. During phases B and C, each training session will be associated with phonemic analysis training and a reading and writing task. At inclusion, a speech and language, psychological, and neuropsychological assessment will be performed to define the four patients' profiles. Patients will be assigned to the different baseline lengths using a simple computerized randomization procedure. The duration of the phases will be counterbalanced. The study will be double blinded. A weekly measurement of phonological and reading skills will be performed for the full duration of the study.

**Discussion:**

The purpose of this protocol is to observe the evolution of reading skills with each type of intervention. From this observation, hypotheses concerning the remediation of developmental dyslexia in HIP-DD children can be tested. The strengths and limitations of the study are discussed.

**Trial registration:**

ClinicalTrials.gov, NCT04028310. Registered on July 18, 2019. Version identifier is no. ID RCB 2019-A01453-54, 19-HPNCL-02, 07/18/2019.

## Background

Studies on learning disabilities in children with high intellectual potential (HIP) are underrepresented in the scientific literature, although their number has been increasing gradually over the years. However, the high prevalence of HIP children in the school-age population, approximately 2.3% of French school children [1], and the high rate of comorbidity with learning disabilities has increased the demand for speech and language therapy care. Some studies claim that the prevalence of HIP among dyslexic readers is higher than the prevalence of HIP among normal readers. For instance, Toffalini [2] and al. reported a proportion of 5.06% HIP children among dyslexic readers compared to 1.82% HIP children among normal readers. Pradeille [3] indicates a proportion of 10% HIP children within a sample of 209 dyslexic readers. According to Winner [4], dyslexia is the most frequent developmental disorder associated with HIP. However, the diagnosis and rehabilitation of children with a high intellectual potential and developmental dyslexia (HIP-DD) remains difficult due to the co-existence of both cognitive features.

Individuals with high intellectual potential represent a small percentage of the population whose intelligence quotient (IQ) is higher than 130 on the Weschler intelligence scale, Wisc IV [5]. However, some authors question the total IQ measure as the only diagnostic tool for these individuals [5–10]. Indeed, the diagnosis of high intellectual potential cannot be limited to the measurement of their IQ, but must also consider the specific characteristics of their brain’s operation. Besides the high IQ score, an HIP child has specific intellectual characteristics, as well as a different cognitive organization [11, 12]. Some authors discuss the presence of specific psycho-affective characteristics in HIP individuals, but there is no consensus on this topic in the literature. Despite this, there are objectively detectable differences on a purely cerebral level. The cerebral particularities of HIP children have been identified in many neuroimaging studies. An increased transmission of information between the left temporal and left central regions, between the left temporal and left parietal regions, and between the left central and left parietal regions has been demonstrated in individuals with HIP compared to neurotypical individuals during a scientific hypothesis generation task [13]. Thus, there appears to be a different distribution of brain activity in HIP individuals, who seem to more efficiently distribute the cognitive resources needed for hypothesis generation [13]. The brains of HIP individuals also differ from neurotypical ones in their strength; there is greater activation of certain brain areas in HIP children during certain cognitive tasks, especially in the prefrontal cortex, the anterior cingulate cortex, and the posterior parietal cortex [14]. In 2021, Christoph Fraenz demonstrated that the strength of neuronal connections at rest between frontal and parietal regions correlates with performance in reasoning tests such as those used in IQ tests [15]. Thus, a higher IQ may mean better quality connections between frontal and parietal regions. Differences in connection between the two hemispheres of the brain were also found in the 2007 study by Luders et al. who found a link between corpus callosum thickness and intelligence scores in adulthood [16]. In addition, numerous studies demonstrate a brain volume proportional to IQ, with a consistent increase in volume as IQ increases [17]. Regarding possible emotional and psycho-affective particularities, there is no consensus in the scientific literature.

Concerning developmental dyslexia (DD), the DSM-V defines it as a severe and persistent disorder that affects reading acquisition, despite a normal intelligence and the absence of neurological or psychiatric pathology, visual and auditory sensory deficits, or socio-educational deficiencies [18]. Developmental dyslexia is a neurodevelopmental disorder that disturbs the automatization of associations between written linguistic units (graphemes) and oral linguistic units (phonemes) [19]. This alphabetic decoding disorder leads to an identification disorder of written words, which can have severe consequences on school learning, on daily and professional life, and on the self-esteem of the patients. The phonological causal hypothesis is currently the most widespread [20]. However, over the last few years, the single cause theory has been criticized in favor of a multi-factorial causal theory [21–23] involving multiple underlying cognitive deficits (UCD) [24, 25]. The two types of UCD most described in the literature are audio-phonological deficits and visuo-attentional deficits. In 2019, Ziegler et al. [26] acknowledged the multifactorial nature of dyslexia but concluded that phonological deficits tended to prevail over the other types of UCD. According to Saksida's study [20], in the non-HIP dyslexic population, the most frequent UCD concerns phonological awareness (PA) and rapid automatized naming (RAN). PA is a cognitive process that allows individuals to manipulate language syllables, rhymes, or phonemes [27]. In the dyslexic population, it is mainly the manipulation of phonemes that is affected [28] and is thus referred to as phonemic awareness. RAN refers to the rapid retrieval of phonological information from a word presented as a picture, followed by its immediate oral production. RAN and phonological awareness are two distinct phonological processes directly involved in dyslexia [29]. In a more recent study [30], phonological awareness was identified as a predictor of word decoding skills and RAN as a predictor of word recognition and reading comprehension. Other researchers agree that the phonological processing deficit in dyslexia is the result of a more fundamental deficit in the perceptual processing of auditory information. Indeed, some dyslexic readers have an allophonic perception of speech sounds which leads to a poor discrimination of acoustic differences, a perceptual deficit in phoneme categorization and consequently a phonological processing deficit. Increased sensitivity to phonemic contrasts between variants of the same phoneme seems to lead to disorders in phoneme categorical perception (CP) [31]. Thus, instead of perceiving sounds in phonemic units, dyslexic readers could perceive them in allophonic units [32].

Concerning HIP-DD diagnosis, the DSM-V reports that compensatory strategies implemented by HIP-DD children may delay the onset of reading disorders and therefore prevent early diagnosis. Indeed, the cognitive profile of HIP-DD children seems to differ from that of HIP-normal readers, but also from that of dyslexic children without HIP [33, 34]. Their double cognitive particularity, HIP and dyslexia, often complicates the interpretation of scores obtained on IQ subtests, but also the interpretation of scores obtained on diagnostic tasks for DD. HIP-DD children, because of their reading difficulties, rarely reach the cut-off score of 130 IQ points determined as an indicator of high intellectual potential. For some authors, it is even meaningless to calculate an IQ when the patient has dyslexia [33]. Similarly, because of their HIP, dyslexic readers sometimes obtain non-pathological scores on the reading and writing tasks used to diagnose dyslexia [35, 36]. The diagnosis of HIP DD children is made difficult by reading and spelling performances that fall between the performances of non-HIP normo-readers and the performances of non-dyslexic HIP [35]. The diagnosis of dyslexia in an HIP child is therefore delayed because of performances that are considered normal by some tests that were not standardized on an HIP population. With their protective factors, HIP children should score above average on some subtests. Thus, when their scores are only average, it often means that their results are actually below what should be expected of them. Several authors describe a phenomenon of "discrepancy" [37, 38], in which dyslexia overshadows HIP and HIP masks dyslexia [33]. So, the cognitive profile of HIP DD is characterized both by phonological deficits related to dyslexia and by often higher verbal and visual-spatial working memory skills, as well as richer vocabulary and grammar than non-HIP readers [34]. Moreover, the study conducted by Van Viersen [35] asserts that the HIP-DD children's UCD primarily concern RAN and phonological awareness. Targeted interventions on RAN or phonological awareness significantly improve reading skills in dyslexic populations [39]. We also know that phonological awareness training combined with print knowledge is more effective than phonological awareness training alone [40, 41]. The effects of RAN training on reading performances have also been proven in a study published in 2019 [42]. Also, an intervention on categorical perception of French phonemes has shown effects on phonemic awareness and reading skills in dyslexic readers [43]. However, remediation methods focused on PA, RAN and CP have not been tested on the specific population of HIP-DD children. Given the large number of differences in brain function between HIP and non-HIP, it would therefore seem appropriate to conduct more studies to improve the therapy of these "twice-exceptional" children. This term refers to individuals with a double particularity; on the one hand, a high intellectual potential, and on the other hand, a comorbid learning disability [44]. Thus, the purpose of this study is to present a single-case experimental protocol proposing an intervention on phonological processes in dyslexic children with high intellectual potential. The detailed methods of this intervention are derived from an ongoing group study evaluating the effectiveness of a UCD intervention on dyslexic children aged 8 to 13 years [45]. The training programs described in this protocol are tailored to the cognitive profile of each participant and can therefore be applied to HIP-DD children.

## Methods and design

### Study design choice

To meet our objectives, we propose an experimental protocol in the form of a single-case multiple-baseline design (A^1^-B-C-A^2^) across 4 patients. Phase A^1^ represents the baseline, which constitutes the patient's initial state, and during which the participant attends standard speech therapy sessions weekly, without intensive training. Phases B and C represent the intervention phases in which remediation is performed [46]: categorical perception (CP) and rapid automatized naming (RAN) are each associated with a phonemic analysis (PA) task. Finally, as it is impossible to return to the A^1^ baseline because of the expected ongoing benefits of the intervention, the A^2^ phase represents a post-intervention phase. During the A^2^ phase, the patient no longer undergoes intensive training but continues to attend standard speech therapy sessions, and the judgment criteria continue to be measured [47]. In cognitive rehabilitation studies, the multiple-baseline single-case experimental design is considered particularly suitable [48]. Several patients with similar cognitive profiles are assigned the same protocol, but the baseline duration differs between them. This additional control ensures that the effect obtained in the B or C phase is attributable to the intervention and that the baseline trend would remain stable in the absence of intervention, which excludes a possible temporal and/or session number bias [49]. The interventions are combined in the form A-B-C without any intermediate baseline return in order to compare the separate effects of each intervention on the studied variable [47]. To control the independent effectiveness of both the B and C interventions [49], a crossover between patients will be achieved by changing the order of interventions. Thus, with four patients, the training order will be counterbalanced so that two of the patients start with PC training, and the two others start with RAN training.

### Recruitment and study population

#### Population

Four French children with a diagnosis of developmental dyslexia and HIP will be recruited for this study. Participants will be enrolled by the principal investigator in the context of her clinical practice. Patients will therefore be informed of the possibility of participating in the study during their regular speech and language therapy appointments. After clarifying the protocol, the speech and language therapist will provide information sheets for the parents and children. A slide presentation will be shown to explain in a simple and entertaining way how the intervention can modify the cognitive processes involved in HIP-DD. Participants and their legal guardians will sign an information sheet and complete an informed consent form before entering the protocol. The inclusion and exclusion criteria are presented in Table 1.

**Table 1:** Inclusion, non-inclusion, and exclusion criteria

Inclusion criteria	Non-inclusion criteria	Exclusion criteria
- Age ≥ 8 years and ≤ 13 years - Diagnosis of HIP confirmed by standardized tests and neuropsychological expertise - Diagnosis of dyslexia confirmed by performance ≤—1.5 standard deviations on leximetry tests compared to the child's developmental age - Performance ≤ -1.5 standard deviations from the norm on phonological tasks in phonological tests - Ownership of a personal computer and an internet connection - Signature of an informed consent form by the parents or legal guardian - Social insurance coverage	- Intellectual deficiency - Neurological Disorders - Pervasive Developmental Disorder - Primary Sensory Impairment - Educational deficits - ADHD - Specific oral language disorders - Previous exposure to intensive daily phonological training - Bilingualism	- Non-compliance with the protocol - Withdrawal from the experiment upon request of the subject or his/her legal guardian

#### Pre-test and recruitment

The four participants will be matched in age and will have a similar cognitive profile. This will be achieved by conducting an inclusion assessment consisting of a complete set of tests assessing written language and underlying cognitive deficits of reading (see Table 2). This assessment will take place in three one-hour sessions. The inclusion assessment will determine the participants' selection before the A^1^ phase. The assessment procedure is the same as for the group study described in the Harrar-Eskinazi et al. study [45].

**Table 2:** An overview of the assessment batteries [45]

Cognitive Processes		Measures	Software
Reading and spelling assessments	Reading aloud	Meaningless text reading	Alouette©
		Meaningful text reading	Evaléo©
		2-min word reading	Evaléo©: Eval2M
		Regular, irregular, pseudo-word reading	Evalec©
	Reading comprehension	Multiple choice statements	Orlec 3©
	Spelling	Phonetic, lexical and grammatical spelling	Chronosdictées©
Underlying cognitive process assessments	Phonological process	Phonological analysis	Evalec©
		Phonological short-term memory	Evalec©
		Rapid automatized naming	Evalec©
	Visual-attentional process	Visual-attentional span	Evadys©
		Global/local analysis	Sigl©
Complementary assessments	Span memory	Digit span	Evaléo©
		Visual-spatial span	Corsi©
	Oral language	Vocabulary and syntactic comprehension	Evaléo©
			E.co.s.se©

### Reading and spelling assessment

#### Reading aloud

##### Non-significant text

*Alouette*© [50, 51] is a test (265 words) considered as the *gold standard* of leximetry tests. It evaluates speed and accuracy when reading a meaningless text. This test provides a reading age [50], a reading speed score, a reading accuracy score, and a combined accuracy and speed index, called reading efficiency [51].

##### Significant texts

*La Mouette*, *Le Pingouin* (*Evaléo* 6–15© [52]): These two meaningful texts (n = 450 words) are equally balanced in terms of word and sentence length, lexical frequency, and syllabic and phonemic complexity to control a retest effect. Reading time and reading accuracy are measured. The maximum reading time is 2 min.

##### Two-minute word reading

*Eval2M* (*Evaléo* 6–15© [52]): This test (*n *= 263 words) assesses the percentage of words presented in 10 columns and ordered based on length and frequency correctly read within a limited time of 2 min.

##### Regular, irregular and pseudo-word reading

*Evalec*© [53]: This computerized test displays the words that need to be identified individually on the screen. The unique feature of this test is to measure the time needed to correctly read words using voice detection. The lexical or sublexical reading processes are assessed by calculating the latency time of correctly read items in msec and the error percentage when reading regular words (*n* = 36), pseudo-words (*n* = 36), and irregular words (*n *= 36).

##### Reading comprehension assessment

The *ORLEC L3* test [54–56] assesses word decoding speed and sentence comprehension. This test presents sentences that need to be finished (*n* = 36) with a word chosen from 5 suggested words. The raw score corresponds to the number of correct items completed in 5 min.

##### Spelling assessment

*Chronosdictées*© [57]: Two dictations of sentences ("A" and "B" for test and retest) are proposed to assess lexical, morphosyntactic and phonetic spelling for each grade of primary and secondary school. Results are given in number of phonetic, lexical, and grammatical errors and in number of segmentation errors and word omissions.

### Underlying cognitive process assessment

#### Phonological processes

The software used for all these tasks calculates a speed and an accuracy score (*Evalec*© [53]).

##### Phonological short-term memory

The pseudo-word repetition task assesses the phonological short-term memory and is composed of pseudo-words of simple consonant/vowel (CV) syllabic structure (*n* = 12) and of pseudo-words of complex consonant/vowel/consonant (CCV) syllabic structure (*n* = 12), from 3 to 6 syllables.

##### Phonological analysis

The task of removing the first syllable from trisyllabic pseudowords (*n* = 10) assesses phonological analysis (e.g. coluti/luti). Two tasks of first phoneme removal from monosyllabic pseudo-words (*n* = 24) assess phonemic analysis (e.g. baf/af and tru/ru).

##### Rapid Automatized Naming

The color naming task assesses rapid automatized naming. Two formats are presented: a matrix of visual color (*n* = 54) and a matrix of written color names (*n* = 54) displayed in 9 lines of 6 colors in a random order. Three colors have a CVC syllabic structure in French (*rouge, jaune, vert*) and three colors have a CCV syllabic structure in French (*bleu, blanc, gris*).

#### Visual-attentional span

The visual-attentional span is measured by a global report task and a partial report task (*Evadys*© [58]). In the global report condition, the subject has to name a sequence of five consonants immediately after the sequence disappears from the screen. In the partial report condition, a vertical line appears and indicates the position of the letter to be named among the five letters displayed. The letter sequences are assembled to avoid activating any memorized lexical knowledge and to prevent any perceptual crowding. An isolated letter identification task is presented beforehand in order to exclude a letter recognition disorder. The software calculates a score in number of successful sequences and a letter span.

#### Global or local visual analysis

*SIGL*© software [59] assesses the ability to focus the attention on a global or on a local visual information analysis mode. The stimuli are hierarchized drawings displayed during 175 ms. The software calculates the gap of performance between the control condition and the interference condition in order to assess the local and global interference. Results are given in response times and in error percentages. To determine the interference asymmetry, the local interference effect is subtracted from the global interference effect.

### Complementary assessments

#### Memory span

##### Digit span

Verbal memory is assessed by a repetition task of 2 to 7 numbers in forward (short-term memory) and in backward (working memory) order (*Evaléo* 6–15©, [52]). The digit span is determined by the number of correctly repeated numbers.

##### Visual-spatial span

The Corsi block-tapping test (*CORSI*© [60]) consists in reproducing the sequence in which the clinician points to different cubes, in the same or in reverse order. The number of cubes tapped in the sequence is progressively increased to determine the visual-spatial span.

### Oral language assessment

If the oral language of the participant has not been assessed previously, three measures will be taken to assess lexical stock and morphosyntactic oral comprehension. The image naming task (*Evaléo* 6–15©, [52]) assesses the participant’s lexical stock of known words and the naming latency time. The image/word association (*Evaléo* 6–15©, [52]) assesses the comprehension’s lexical stock. The image/phrase association (*E.CO.S.SE*, [61]) assesses the syntactic-semantic comprehension.

### Procedure

**Fig. 1:**
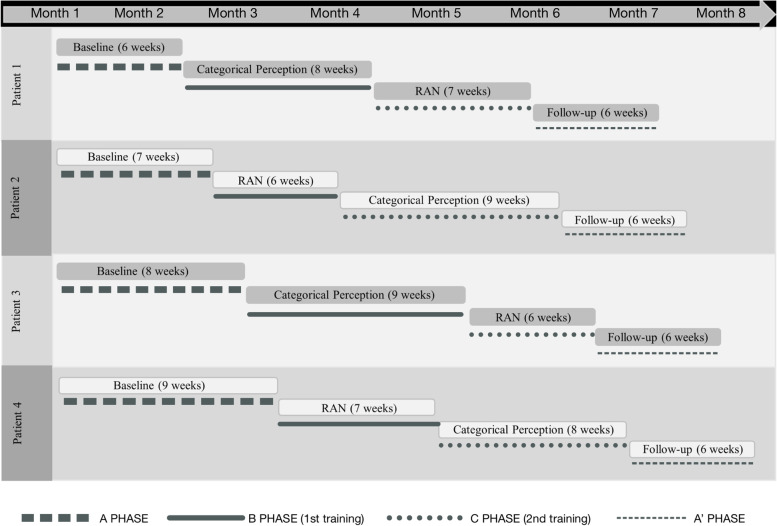
Overview of participants’ timeline. RAN: rapid automatized naming

For this multiple-baseline A_1_BCA_2_ design across four participants, A_1_ phase corresponds to the baseline, where the participants follow a standard speech therapy remediation program. This standard remediation corresponds to a speech and language therapy intervention without daily training. The speech and language therapists target the symptoms and pathological behavioral manifestations of dyslexia without specifically working on the underlying cognitive processes. Both the B and C phases correspond to two distinct types of audio-phonological training, respectively categorical perception (CP) training, and rapid automatized naming (RAN) training. During both the B and C phases, each training session is coupled with phonemic analysis (PA) training and a reading and writing task. A_2_ phase represents the post intervention phase where the participants stop the intensive training but continue to follow a standard remediation program. The detailed timeline of the participants is presented in Fig. 1. To obtain enough data for statistical analysis, each phase of this protocol involves at least 6 measurement sessions [62] held on a weekly basis. Phases B and C will be introduced in a staggered timeframe across participants. In other words, each participant's baseline will vary in length and number of measurement points. Thus, with one measurement session per week, Participants 1, 2, 3 and 4 will respectively begin the intervention after a baseline period of 6 weeks (6 measurement sessions), 7 weeks (7 measurement sessions), 8 weeks (8 measurement sessions), and 9 weeks (9 measurement sessions). The two training phases are designed to avoid any confounding values of time or number of sessions. The follow-up phase (A_2_) will also be introduced sequentially but will last 6 weeks (6 measures) for all participants. As a result, the total duration of the four phases will not exceed 30 weeks. Throughout the entire protocol, patients will continue standard weekly speech therapy sessions with their speech and language therapist. Measurement sessions will be performed at the end of each of these sessions by the principal investigator.

### Objectives

*The main objective* is to compare the effect on the phonological abilities of HIP-DD subjects of an intensive intervention combining training in categorical perception (CP), rapid automatized naming (RAN), and phonemic analysis (PA) versus a standard remediation program, at the end of the training program and at 6 weeks post-training.

*Main Hypothesis:* We assume that intensive training coupled with standard dyslexia remediation will significantly improve participants' phonological skills.

Two secondary objectives were identified:

- *Objective 2*: To compare the effect on reading efficiency of an intensive intervention on CP, RAN, and PA versus standard remediation, at the end of the training program and at 6 weeks post-training.
- *Hypothesis 2*: We hypothesize that intensive CP, RAN, and PA training significantly improves reading skills at the end of the training period and 6 weeks after.
- *Objective 3*: We explore the effect of the sequence of CP and RAN training phases on participant’s phonological and reading skills.

### Intervention

#### Remediation protocol

In phase A, the standard speech therapy session for dyslexia contains grapheme-phoneme conversion exercises, lexical spelling tasks, and phonological awareness tasks without visual support. According to the NGAP [63], the session should last at least 30 min.

During the B and C intervention phases, the children continue standard speech therapy sessions (as in phase A) and add 15 min of daily training at home, 5 days a week. The main experimenter organizes a practice training session with the legal guardians and patients at the beginning of the protocol to ensure that the participants understand how to complete the training sessions correctly without any assistance. The first type of intervention corresponds to a 10-min training session on categorical perception using Rapdys© [43]. The second type of intervention is rapid automatized naming and lasts 10 min. Both interventions (RAN and CP) are systematically combined with 5 additional minutes of phonemic analysis. On the two days off each week, the child does not perform any training.

#### Descriptions of specific interventions

##### Categorial perception—rapdys©

Categorical perception training is carried out at home: 10 min per day, 5 times per week for the whole duration of the phase using RAPDYS© [43]. This software proposes a series of training sessions allowing the patient to discriminate more and more finely between two phonemes with different voicing (e.g. /d/ and /t/). The stimuli used depend on the level of difficulty: 5 levels based on the difference in VOT (Voice Onset Time) between the stimuli. Training consists of two tasks: identification and discrimination. In the identification task, the participant listens to a sound stimulus and has to determine which phoneme was heard. In the discrimination task, the participant hears two phonemes in a row and has to say whether they were the same or different.

##### Rapid automatized naming—naming speed

**Fig. 2:**
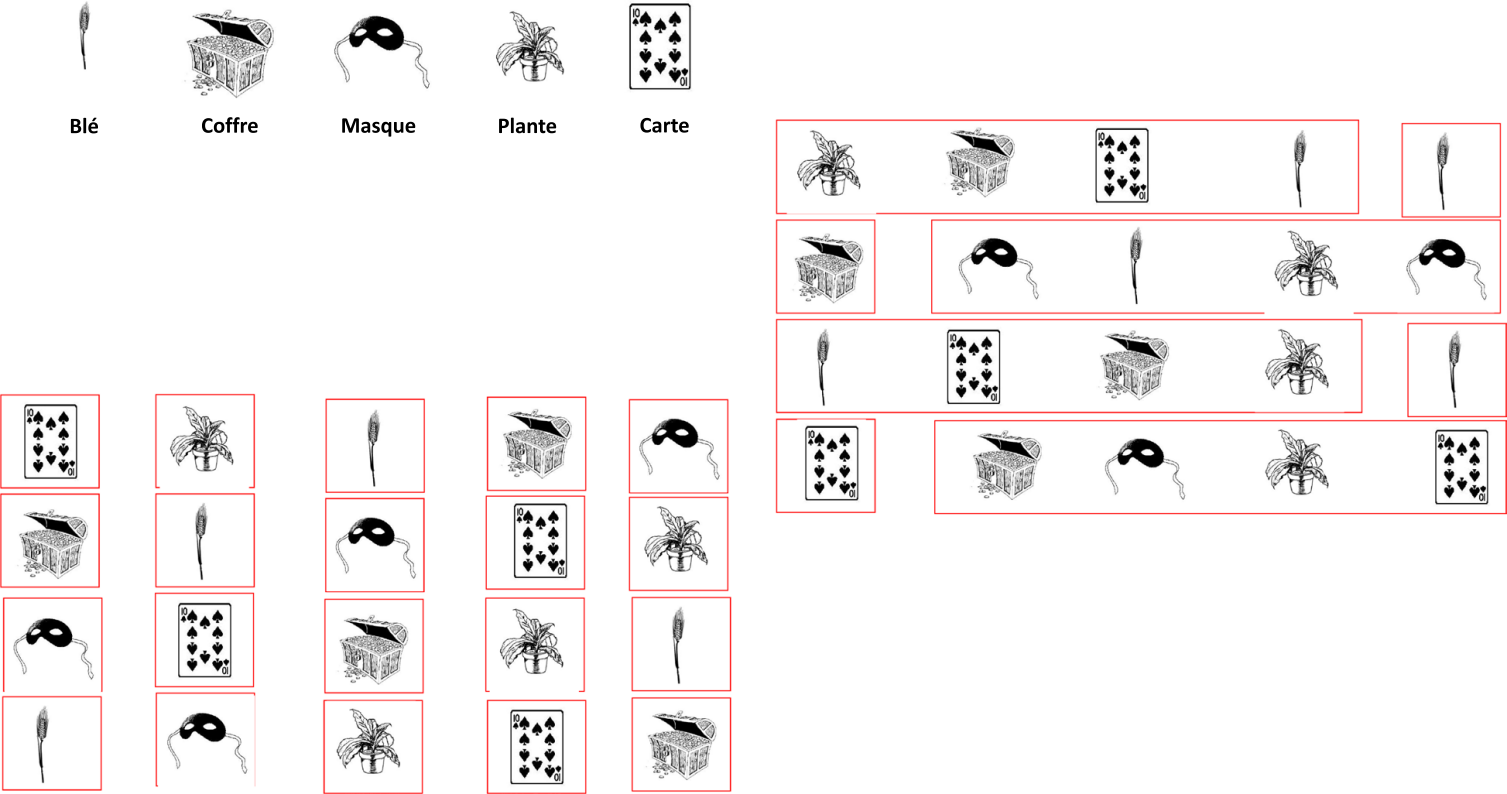
An example of the RAN board and the preliminary practice line

The rapid automatized RAN training is performed at home: 10 min per day, 5 days per week for the whole duration of the phase, using the Naming Speed program. This program was created by Karine Harrar Eskinazi, Julie Nothelier, and Marine Versio for the needs of the forthcoming study "Developmental dyslexia and method of remediation (DDMR): Multimodal intervention in French children aged from 8 to 13 years'' [45] because no other software for rapid naming training was available in French. This program was inspired by the Italian software "Run the RAN" [42]. Five black and white drawings of objects from the LEAD lexicon database [64] are displayed on the screen and repeated on horizontal lines randomly displayed in boards of 20 to 60 stimuli. The patient must name, as quickly and accurately as possible, all the images presented, from left to right (in the direction of reading) following an imposed cadence; a red frame is automatically displayed on the screen and gives the patient the naming rhythm. A single image is framed at first, and as the training progresses, the frame surrounds more images (up to 5) and the naming speed increases (from 200 ms/item to 50 ms/item) (Fig. 2***)****.*

##### Phonological analysis—phoneme fusion and segmentation (in both the b and c phases)

During both training phases (PC and RAN), a phonemic analysis task is associated with the daily exercises. This task combines two processes: phonemic segmentation and phonemic fusion. Phonemic fusion consists in merging phonemes pronounced in oral form; for instance, the child hears the phonemes /p/-/i/-/r/-/õ/ one after the other and has to fuse the phonemes together in order to pronounce the logatome "*piron*". The segmentation task is the exact opposite: the child hears the logatome and must segment it into phonemes. In order to train the grapheme-phoneme conversion processes, the child is asked to read the ten items at the end of the training session, and to train the phoneme-grapheme conversion process, he must also write them. The logatomes are computer-generated. Participants are asked to fuse ten logatomes per day after each categorical perception training and to segment ten logatomes per day after each RAN training.

### Outcomes

**Fig. 3:**
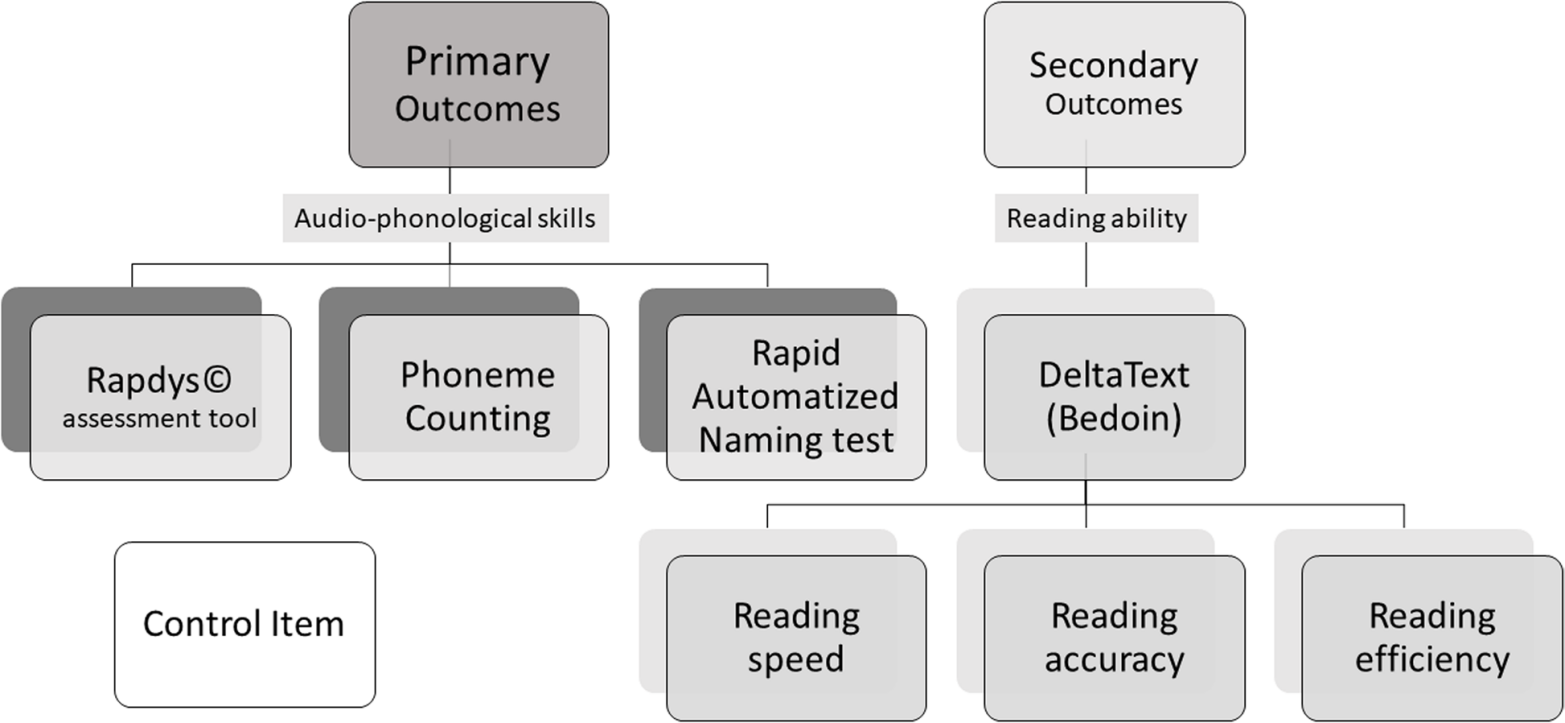
Weekly measurement session for each participant during the entire protocol period

The two primary judgment criteria are each evaluated by three different measures. In order to meet the internal and external validity criteria of the Risk of Bias in N of 1 Trials (RoBiNT) scale [65], the outcome measures are assessed once a week throughout the entire protocol, from the beginning of phase A_1_ to the end of phase A_2_. The measurement session is performed at the end of each weekly speech and language therapy appointment (Fig. 3)*.*

#### Primary outcomes

The first outcome is a performance gain in audio-phonological skills. It is evaluated by three distinct measures as recommended by Tate et al. [66]:

##### Rapdys© Assessment

Rapdys© [43] is a program designed to assess and improve the discrimination of voicing boundaries of the phonemes of the French language (opposition of muted and voiced consonant sounds, for example /b/ & /d/). The integrated evaluation software provides an assessment of the child's perceptual system and thus an objective measure of potential progress achieved during training. According to the authors, there is no test/retest effect since the stimuli are presented in a random order and no feedback is provided. Studies conducted did not show any increase in perceptual performance in the control group when the evaluation task was repeated at regular intervals. The score is the percentage of correct answers and will be the judgment criterion for this measure.

##### Phoneme counting

To assess phonological analysis skills, a list of 10 logatomes is presented to patients from an oral input. The examiner reads the logatome to the child and asks for a count of the phonemes composing it. For example, for the logatome /pabou/, the participant has to segment the phonemes and count them: /p/-/a/-/b/-/ou/ = four phonemes. The child has to answer four. The logatome lists were generated from the free software *Logatron* [58]. In order to obtain 10 different logatomes in each of the 30 repeated measurement sessions, a list of 300 bisyllabic logatomes was generated. The logatomes in this list follow the phonotactic rules of French. Some of them contain complex phoneme groups (consonant clusters) and others are simpler. If the child makes a mistake, the item is presented again until it is successfully answered. The time (in seconds) is measured and used as a judgement criterion.

##### Ran colors

**Fig. 4:**
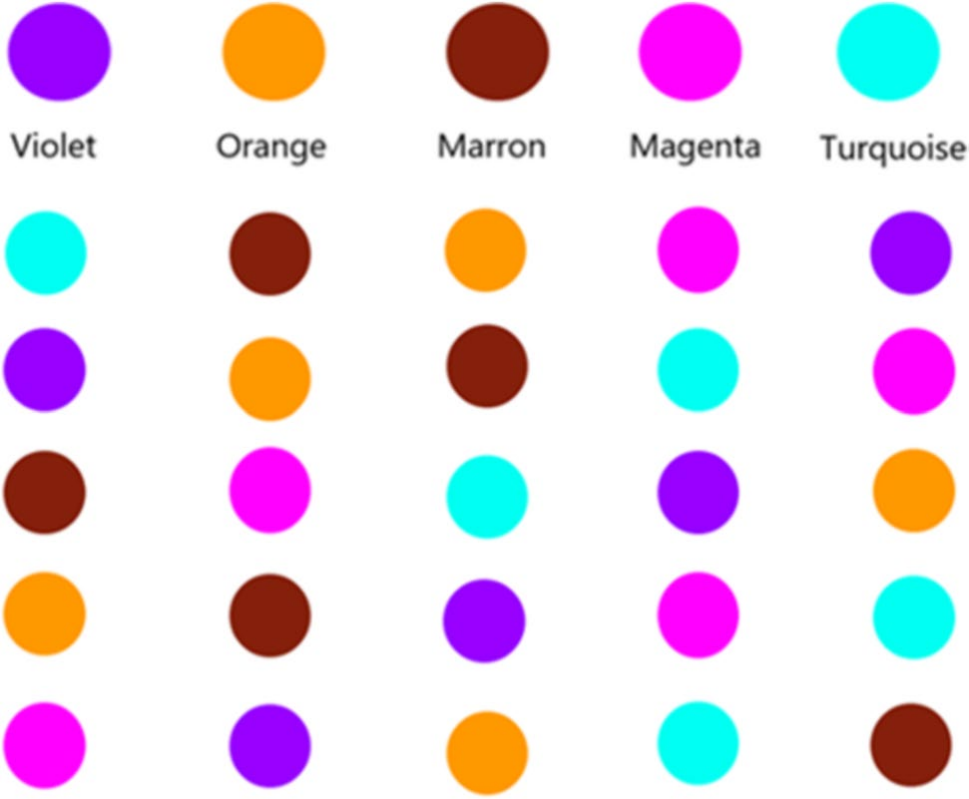
An example of a test board next to the preliminary practice line

To measure rapid automatized naming skills, a board of 54 colors (9 lines of 6 colors) is presented in a random order. Its organization is based on the initial “Naming Speed” board [45] but with more complex colors (purple, orange, turquoise etc.) displayed in a different computer-generated random order for each measurement to reduce the learning effect. The participant must name all the colors on the board in the direction of reading (from left to right) and as quickly as possible while making as few mistakes as possible. The raw scores collected are the time in seconds and the number of errors per board. An accuracy score is calculated by dividing the number of correctly named colors by the total number of colors on a board. A second score is computed by dividing the time spent naming the entire board by the number of correctly named colors. An example of a test board is shown in Fig. 4 next to the preliminary practice line.

#### Secondary outcomes

The generalization measure –the measure of reading efficiency– is also assessed using three different scores, all from the "*DeltaText*" leximetry test (*Bedoin*, 2017).

The *DeltaText* consists of four meaningless texts matched for word count and syntactic, lexical, and phonological difficulty. Each text is composed of 201 regular words. The maximum reading time is set to 3 min. During the test, the number of errors and the number of words read at the end of 1 mn 30 and at the end of the 3 min are recorded. The instruction given to the patient is to read as quickly as possible and with the fewest mistakes as possible. If the 201 words are read before the end of the allocated time, the reading time is recorded. Three measures are obtained:

##### Reading speed

The reading speed (in number of words read per minute).

##### Reading accuracy

The reading accuracy (in percentage of words correctly read).

##### Reading efficiency

The reading efficiency: CTL = [C *(number of words read correctly)* / TL *(child reading time)*] x 120 s *(maximum reading time)*].

#### Control item

**Fig. 5:**
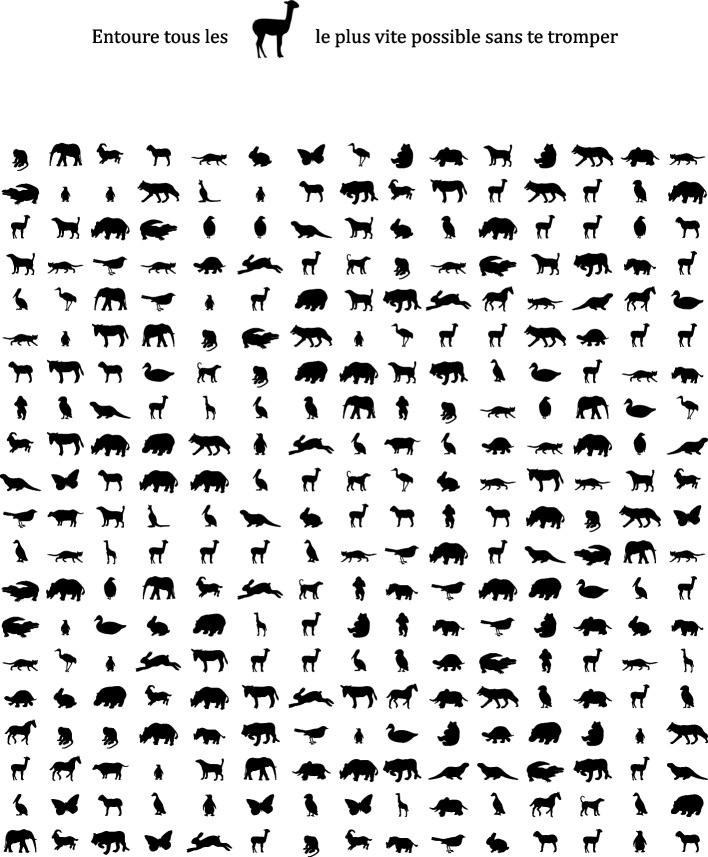
Example of a control item board with the instructions for the participant in French

To control the specificity of the training, a control test is also proposed, in which non-linguistic target symbols must be circled among visual distractors. A board contains 300 items, including 30 targets. The participant has to find all the targets among the 270 distractor animals on the board during a given time. The board is printed in A4 format, and the participant has 60 s to circle as many targets as possible as fast as possible without circling the distractors. For each measurement session, a new board is randomly generated in a different disposition through a computerized process. An example of a board is shown in Fig. 5.

### Randomization allocation and blinding

To introduce a single-blind effect, the children will know what their training program involves but not the expected effects. To ensure double blinding, the speech therapist will be aware of the experimental phases the children are in, but will not intervene in the measurements nor in the collection of results. The members of the experimental teams will not know which phase the children are in, and will simply collect the measurements during the weekly measurement session. This will maintain double blinding.

The internal validity of the single-case experimental design (SCED) is increased when patients are assigned in an unbiased manner to the baseline duration [67]. Thus, patients will be randomly assigned to the different baseline lengths using a simple computerized randomization procedure. The duration of the intervention phases is also determined in a counterbalanced manner. Two of the four participants will begin with categorical perception training and the other two will begin with rapid automatized naming training. This order follows the baseline durations assigned at the beginning of the experiment: the number of weeks per phase is calculated to avoid confounding variables. The allocation of participants to the 4 designs (type of intervention and length) is thus randomized.

### Training supervision and treatment integrity

The daily training sessions are carried out at the patient's home, and the parents or legal guardians are asked to fill in protocol monitoring forms. These forms contain both simplified training instructions for the parents and charts to record the child's scores each day. However, the weekly speech and language therapy sessions also provide an effective way to ensure proper completion of the protocol and compliance with the training instructions. Therefore, these records will be checked at each weekly speech and language therapy appointment to confirm that the training program is correctly followed. The RapDys© software will also provide a record of the dates and results of each training session. The measurements will be assessed by a member of experimental teams after each weekly speech therapy session.

### Confidentiality

All computerized experimental data from the study will be stored on a password secured network only known by the investigators. Paper data will be kept in a locked case. Patients will be anonymized when data are published.

### Refusal of study participation and drop-out

Consent for study participation is obtained at the pre-inclusion assessment and no justification is required in case of refusal. Withdrawal from the protocol is possible at any time at the request of the patient or legal guardian and in case of non-compliance. The criteria for exceptional discontinuation or modification of the protocol are withdrawal of consent to participate, a medical condition, or any other event involving a non-compliance.

### Adverse events

There are no predicted adverse or dangerous events for the participants in this study.

### Intended descriptive and inferential statistical analyses

First, a visual and descriptive analysis will be performed (Glass’∆ and Cohen’s d [68]). Then, we will use the Tau-U analysis [69], which measures effect size for single-case studies. The baseline trend could be controlled by the Baseline Corrected Tau [70].

To fit the design, missing measurement sessions will be considered as missing data and will not be imputed. Also, according to James E. Pustejovsky, we will make available the raw data used for effect size calculations, so that other researchers can easily replicate and extend our analyses [71].

## Discussion

This paper presents an experimental protocol for a single-case multiple-baseline design across 4 participants to observe the effects of an intensive audio-phonological training program on the phonological and reading skills of four children with developmental dyslexia and HIP (HIP-DD). The lack of data regarding the remediation of HIP-DD children forces clinicians to use non-specific remediation methods for twice-exceptional children, which are often not very effective.

The results of this study, if successful, will provide a partial answer to this problem. Furthermore, the link between the single-case design and Evidence Based Practice (EBP) [72], which is widely used by clinicians in their daily practice, opens a real gateway between the research and clinical worlds. The American Speech-Language-Hearing Association has also recommended the use of EBP in interventions for communication disorders since 2005 [73]. Most speech and language therapists are therefore familiar with the use of baselines and with the within-subject control approach. Thus, if the results are consistent, they can be directly interpreted by clinicians, and the data regarding remediation can be easily adapted and quickly applied in their practice.

Furthermore, small sample studies allow for a detailed qualitative analysis and are a good alternative to group studies when the target population is rare. The single-case design allows a more exhaustive qualitative analysis of the anamnesis and screening data in order to better understand the cognitive language profile of each participant. The staggered configuration of the participants' timeline allows a control of temporal and session number variables. Despite the thorough attention paid to respecting scientific criteria that ensure the highest reliability of the outcomes, some limitations were found when preparing this protocol. To avoid cognitive overload and participant dropout, an intervention targeting all underlying cognitive deficits was excluded. First, the time required for a measurement session would be excessive, as would the total duration of the protocol. Also, since phonological theory is still the most widely believed causal hypothesis of developmental dyslexia [66], an intervention that only targets the audio-phonological processes involved in dyslexia was selected. Thus, only RAN and CP were measured and offered as training in this study. A similar protocol focusing on visual-attentional aspects could be considered at a later stage. If the results are conclusive, a remediation protocol focusing on all underlying cognitive deficits should be tested in a larger sample study without the constraints of repeated measurements induced by the SCED.

In conclusion, the population of HIP-DD patients represents a significant part of speech and language therapists' patient base but remains underrepresented in the scientific literature. Consequently, the therapeutic approaches for these patients are still not sufficiently structured and validated. The results of this study should constitute a starting point for further progress in the remediation of written language disorders in children with HIP, both in the clinical and the research fields.

## Data Availability

This manuscript does not contain any data at this stage, but future data will be made available upon reasonable request by sending a mail to darrot_research@yahoo.com or through a permanent weblink to datasets.

## Abbreviations

HIP: High intellectual potential
HIP-DD: Dyslexic with high intellectual potential
IQ: Intellectual Quotient
DD: Developmental Dyslexia
UCD: Underlying Cognitive Deficit(s)
RAN: Rapid Automatized Naming
CP: Categorical Perception
PA: Phonemic Analysis
CV: Consonant/Vowel
CCV: Consonant/Consonant/Vowel
VOT: Voice Onset Time
SCED: Single-Case Experimental Design
EBP: Evidence Based Practice
